# Dr. Weatherhead's Treatise on Infantile and Adult Rickets

**Published:** 1820-06-01

**Authors:** 


*A Treatise on Infantile and Adult Rickets; with some Remarks appended on Nursing, for the Consideration of Mothers, &c. &c.* By GEORGE HUME WEATHERHEAD, M. D. &c. &c. Small Octavo, pp. 128. London, 1820.

DR. WEATHERHEAD has chosen a hard-hearted, or rather a heartless Patron for his work. He has dedicated it to "the PETRIFIED SKELETON in the British Museum, in A—STON—ISHMENT, at its hardihood and determination to defeat, even after death, any attack of a mollities ossium."

Now, as the work is evidently designed to circulate among Nurses rather than physicians, we apprehend, that the pun in the above eccentric dedication, is somewhat ill-timed as well as ill-placed; for, however inclined the nursing tribe may be to relish a joke, at man's *entrance* into this world, they are the very worst auditors for a pun on the subject of death, and particularly on a human skeleton. We would, therefore, advise Dr. Weatherhead to cancel the said dedication, as we do not think it consistent either with the dignity of a philosophical subject, or the train of reflections which *naturally* arise in the human mind, while contemplating the ghastly memento of our frail and perishable existence!

Those portions of the work which come within "the vow of our ascetic order," are comparatively small, and will not detain us long.

The symptomatology and pathology of Rickets, are too familiar to require delineation here. Dr. Weatherhead's "New Theory of Rickets," is this:—

"I regard the origin of rickets to consist in an inability in the proper decomposing apparatus to neutralize the fluid super-salt, (super-phosphate of lime) and thus fix it as a solid." 86.

To the work itself we must refer for the arguments and facts on which this hypothesis is founded.

Our author's indications of cure are, 1st. To bring the digestive organs to perform their functions well. 2dly. To confirm the strength gained by proper nutriment, by giving tone to the system generally. The good effects of purgatives in this disease, our author thinks, and so do we, "are not limited to that of simply evacuating the bowels; their main efficacy consists in correcting the morbid nature of their secretions." He gives a preference to rhubarb and submurias hydrargyri; and when the patient is convalescent, a saline mineral spring is advisable, particularly that of Leamington in Warwickshire. Emetics our author occasionally employs —"with the object of *dejecting* the sympathy they excite to the bowels, and *ejecting* it on the surface." The deficiency of *phosphate of lime,* as the proximate cause of rickets, has induced some physicians to try the efficacy of this earth; but the trial, says Dr. W. was futile. We might as well "cram quartern loaves down the gullet of the Farnese Hercules," "without an internal apparatus to digest them." Of all the remedies given with a local object, our author has found none so pre-eminently useful as the alkalis. "I was induced to try their effects from reading Brandish's book, and the result has been, in several instances, most happy." He objects to the unscientific mode of administering the medicine, pursued by Mr. Brandish, to remedy which, he usually prescribes so many drops of the aqua potassæ, *pro viribus et ætate egroti,* mixed in a stated quantity of aqua calcis, taken in some bland vehicle. The cold bath, and other well known means of correcting a cachectic state of body, are also mentioned by our author. The work concludes with a description of a new reclining couch, of which the author gives a neat engraving. Here too Dr. Weatherhead cannot restrain the effusion of his wit, though, as we said before, it is exceedingly out of place. "The reclining board, says Dr. W. in common use is constructed on a *Tyburn principle,* that of suspending the unlucky patient by the neck." These unseasonable sallies may make the vulgar smile, but they will make the sensible grieve. We leave it to our author's discretion which of these two classes it is most desirable to propitiate in our favour.

Such are the leading *practical* features of the little work before us. Dr. Weatherhead, however, is not sparing of ingenious speculation, and learned research. Our author is a great admirer of the ancients. We too can admire them, but as men like ourselves. We have carefully perused the writings of Hippocrates and the principal fathers of physic; and the impression left on our minds is this;—that the Ancients knew *very little* of which we are ignorant—and that the Moderns know *very much* of which the Ancients knew nothing. Moreover, it may be safely asserted that, to get at one truth in the writings of the fathers, we are obliged to wade through ten errors; and when that one truth is found, it is not intrinsically more valuable than if recorded in Thomas's Practice of Physic, or Cooper's Surgical Dictionary. Yet we recommend the study of the ancients—but it is principally to *exercise* the mind; in the same way as we make our sons construe their lessons by aid of the Lexicon and Syntax, rather than by the more easy method of a literal translation.

